# Performance Evaluation Metrics and Approaches for Target Tracking: A Survey

**DOI:** 10.3390/s22030793

**Published:** 2022-01-20

**Authors:** Yan Song, Zheng Hu, Tiancheng Li, Hongqi Fan

**Affiliations:** 1 Key Laboratory of Information Fusion Technology, Northwestern Polytechnical University, Xi’an 710072, China; syzx@mail.nwpu.edu.cn (Y.S.); zheng.hoo@mail.nwpu.edu.cn (Z.H.); 2 Key Laboratory of Science and Technology on ATR, National University of Defense Technology, Changsha 410073, China; fanhongqi@nudt.edu.cn

**Keywords:** performance evaluation, cloud barycenter evaluation, fuzzy CE, grey clustering

## Abstract

Performance evaluation (PE) plays a key role in the design and validation of any target-tracking algorithms. In fact, it is often closely related to the definition and derivation of the optimality/suboptimality of an algorithm such as that all minimum mean-squared error estimators are based on the minimization of the mean-squared error of the estimation. In this paper, we review both classic and emerging novel PE metrics and approaches in the context of estimation and target tracking. First, we briefly review the evaluation metrics commonly used for target tracking, which are classified into three groups corresponding to the most important three factors of the tracking algorithm, namely correctness, timeliness, and accuracy. Then, comprehensive evaluation (CE) approaches such as cloud barycenter evaluation, fuzzy CE, and grey clustering are reviewed. Finally, we demonstrate the use of these PE metrics and CE approaches in representative target tracking scenarios.

## 1. Introduction

Target tracking is widely involved in many problems of significance, such as military defense, automation/driverless transportation, intelligent robots, and so on. There are many outstanding works to provide guidance on the implementation of target-tracking algorithms. For the issue of multitarget tracking, the International Society of Information Fusion (ISIF) took track estimation, data association, and performance evaluation into account many years ago. The textbook [1], which is a tutorial of the known target-tracking algorithms and the class material [2], can assist researchers in re-implementing these methods and developing advanced methods. Some common toolboxes include the Recursive Bayesian Estimation Library (ReBEL) [3,4,5], the nonlinear estimation framework [6,7,8], and the Tracker Component Library [9]. The Open Source Tracking and Estimation Working Group (OSTEWG), which is the working group of the ISIF, revisited the current widely used methods to make up an open-source framework in [10,11], which is named Stone Soup and is available from the website https://github.com/dstl/Stone-Soup/ (accessed on 10 November 2021). Another ISIF working group, the Evaluation of Techniques of Uncertainty Reasoning Working Group (ETURWG), develops the uncertainty description in the domain of target tracking and introduced the Uncertainty Representation and Reasoning Evaluation Framework (URREF) [12] to Stone Soup.

This paper focuses on the performance evaluation (PE) of target-tracking algorithms, which plays a key role in the comparison of existing algorithms and in putting forward a new algorithm. PE is referred to the assessment and the evaluation of various performance metrics of a system [13,14], whose significance lies in providing evaluation results of the system performance [15], as well as the reference basis for the optimization of the system performance.

The basic process required for tracking evaluation is that when both truth targets and tracks are available, the first step is to find an association between the true target and a track so that performance measures can be computed. However, it was assumed that there is a unique association in this paper. The association algorithms can be found in [16,17,18,19]. After the tracks were assigned to targets, we computed the various performance measures to analyze the target-tracking algorithms and optimize these algorithms.

For the practitioner, the PE problems can be divided into two stages. The first is to choose the relevant effective metrics, and the second is to evaluate a single score through these metrics [20]. In this paper, we reviewed the measures to evaluate the performance of the target tracking system. These metrics were grouped into the categories of the correctness, timeliness, and accuracy. The assessed results of each metric were weighted and combined to give an overall performance measure. Further, we designed simulations that employed several PE approaches based on these metrics to illustrate their use in the target-tracking problem.

The rest of this paper is organized as follows. Section 2 introduces general evaluation metrics and sorts categories by the characteristics of each metric. Section 3 reviews classic PE approaches. The simulation results of our approach in the context of target tracking are given in Section 4. Section 5 concludes our work and faces remaining challenges.

## 2. A Classification of the Comprehensive Evaluation Metrics

**Figure 1:**
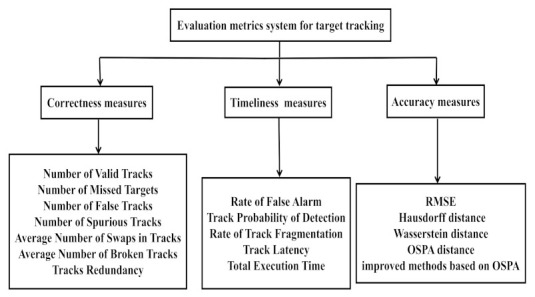
Classification of representative CE metrics.

In the context of target tracking, a variety of evaluation metrics with physical significance have been proposed, which can evaluate the practicability of the tracking algorithm and the consistency of the expected and assessed results. These metrics can be divided into three categories: effectiveness, timeliness, and accuracy, which can be seen in [21,22,23,24]. This paper also followed this division criterion for convenience. What the correctness [18,25,26,27] usually represents is the number of missed/false targets, etc. The timeliness assesses the time performance of the estimated track [23,28], which is a crucial measure for online target tracking. The accuracy metrics can be defined in different ways according to the different scenario requirements, in which the (root-) mean-squared error ((R)MSE) is commonly used in trajectory error (TE), tracking position error (TPE), and tracking velocity error (TVE) [29], and the other accuracy metrics refer to [28]. References [30,31] combined the multiple-object-tracking precision (MOPT) and the multiple-object-tracking accuracy (MOTA) to describe the effectiveness and the timeliness of the multiple-object-tracking systems, but they disregarded the effect of the error. In addition, the measures, such as the cross-platform commonality, the track purity, the processor loading, etc., were considered in [7,26,32,33,34,35,36]. The comprehensive evaluation (CE) metric system is shown in Figure 1.

### 2.1. Correctness Measures

**Figure 2:**
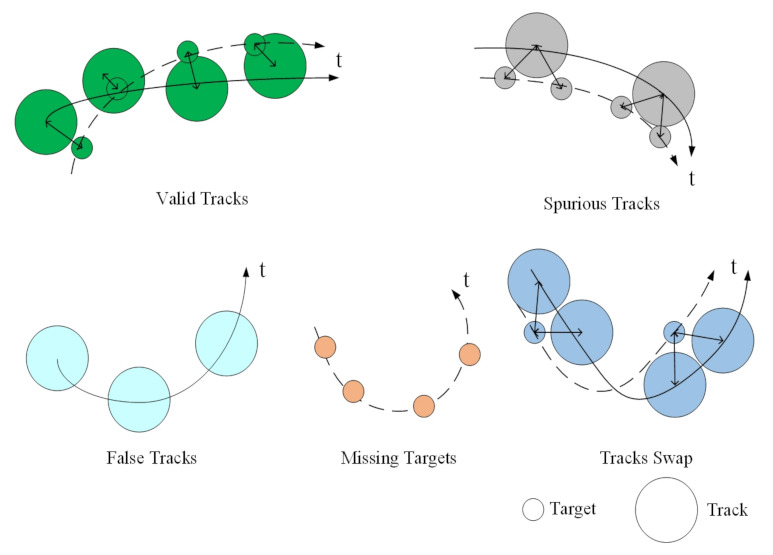
Mapping the tracker hypotheses to objects. In the easiest case, different associations result in evaluation metrics.

The correctness measures the numerical characteristics of the acquired data and calculates how many mistakes the tracker made in terms of misses, false tracks, and so forth, which can be briefly described by Figure 2, where the small and large circles represent the truth target and the estimated track, respectively. The solid and dashed lines denote the trajectory of the target and the curve of the track, respectively. Given a time interval $t\in \left[t_{1},t_{2}\right]$, the correspondence between the target and the track is established in Figure 2. These measures are explained in detail below.

- Number of valid tracks (NVT):
  If a track is assigned to a target and the target has only one track, then the track is validated. $N_{valid}\left(t\right)$ denotes the NVT at time *t*;
- Number of missed targets (NMT):
  A target is missed if it is not associated with any track. $N_{missed}\left(t\right)$ is the NMT at time *t* [26];
- Number of false tracks (NFT):
  A track is false if it is not assigned to any target. $N_{false}\left(t\right)$ denotes the NFT at time *t*;
- Number of spurious tracks (NST):
  A track is defined as spurious if it is assigned to more than one target, and the NST is denoted as $N_{spur}\left(t\right)$;
- Average number of swaps in tracks (ANST):
  Different confirmed tracks may be assigned to a particular truth. This can happen when crossing targets and targets come close to each other. Assume $N_{swap}\left(t\right)$ as the ANST at time *t* [26];
- Average number of broken tracks (ANBT):
  There is also probable that no track is assigned to the truth for several time steps. If there is no assigned track to the truth, the number of broken tracks is counted at each time step. Reference [26] employed the ANBT to check the track segment associated with the truth;
- Tracks redundancy (TR):
  TR is represented as the ratio of validated tracks and total assigned tracks:
  (1) $$TR\left(t\right)=\frac{N_{valid}\left(t\right)}{N_{valid}\left(t\right)+N_{spur}\left(t\right)}.$$

### 2.2. Timeliness Measures

The performance measure provides more information about the track persistence, which is also an indispensable part of the evaluation metrics [37]. Some timeliness metrics for the PE are given as follows:

- Rate of false alarms (RFA):
  The RFA [38] is defined as the NFT per time step, which can be denoted as follows:
  (2) $$FLR\left(t\right)=\frac{N_{false}\left(t\right)}{t}.$$
- Track probability of detection (TPD):
  In the time interval $\left[t_{1},t_{2}\right]$, assume $t_{first}^{i}$ and $t_{last}^{i}$ as the first and last time that the *i*th target is present, respectively. According to [39], the TPD of each target is represented as:
  (3) $$P_{d}^{i}=\frac{t^{{}^{\prime }}}{t_{last}^{i}-t_{first}^{i}},$$
  where $t^{{}^{\prime }}$ denotes the persistent duration where the *i*th target is assigned to a valid track;
- Rate of track fragmentation (RTF):
  It is likely that the track obtained through some tracking algorithms may not be continuous sometimes. The track segment is assigned to the *i*th truth, the number of changes that the continuous track becomes fragmental is defined as $TFR_{i}$ when the track segment is assigned to the *i*th truth. The smaller the RTF is, the more persistent the tracking estimated by the algorithm is [39];
- Track latency (TL):
  The TL, the delay from the moment that the target arises in the view of the sensor to the moment that target is detected by the tracker in the running period, is a measure of the track timeliness;
- Total execution time (TET):
  The computational cost is another important factor to be considered in the PE of target tracking. Therefore, the total time that is taken to run the tracker is expressed as the TET for each tracking algorithm.

### 2.3. Accuracy Measures

The measure, favored by the majority of researchers, is a primary choice in evaluating the target tracking, in which several measures can be defined as based on the type of distance between the set of truths and tracks:

- RMSE:
  The RMSE is defined in terms of the estimation error $e_{k}$, which is the average difference between the estimated state ${\tilde{X}}_{k}$ and the truth state $X_{k}$, as:
  (4) $$R^{l}\left(k\right)=\sqrt{\frac{1}{n}\sum_{k=1}^{n}{∥e_{k}∥}^{2}},$$
  where *n* denotes the number of the *l*th targets detected at the *t*th time step, ${∥e_{k}∥}^{2}={e_{k}}^{T}e_{k}$. Th MSE/RMSE has long been the dominant quantitative performance metric in the field of signal processing. For the traditional target-tracking algorithms, the aim is to minimize them between the target truth and the estimated track [40], which is not suitable for the track assignments that do not have a one-to-one correspondence. At present, the CE metrics have been widely used in the Hausdorff distance [41], the Wasserstein distance [42,43], and the optimal subpattern assignment (OSPA) distance [20,44,45,46];
- Hausdorff distance:
  The Hausdorff distance is a common method of measuring the distance between two sets of objects, which can be used to measure the similarity between tracks and is given by:
  (5) $$d^{H}\left(X,\tilde{X}\right)\overset{\Delta }{\;=}max\left\{\max_{x\in X}\min_{\tilde{x}\in \tilde{X}}d\left(x,\tilde{x}\right),\max_{\tilde{x}\in \tilde{X}}\min_{x\in X}d\left(x,\tilde{x}\right)\right\},$$
  where $X=\{x_{1},x_{2},\cdots ,x_{k}\}$, $\tilde{X}=\left\{{\tilde{x}}_{1},{\tilde{x}}_{2},\cdots ,{\tilde{x}}_{k}\right\}$. $x_{i}$ and ${\tilde{x}}_{i}$ are the *i*th target and the *i*th track, respectively. $d(x,\tilde{x})$ is the Euclidean distance between *x* and $\tilde{x}$. It has been proven that the Hausdorff distance is very useful in assessing the multitarget data fusion algorithms [47,48,49]. Meanwhile, the distance is relatively insensitive to differences in the numbers of objects;
- Wasserstein distance:
  The Wasserstein metric was initially used to measure the similarity of probability distributions [50] and was proposed for the sets of targets in [51]. The Wasserstein distance between *X* and $\tilde{X}$ is:
  (6) $$\begin{array}{c} d^{W}\left(X,\tilde{X}\right)=\min_{\pi \in \Pi_{n}}\sqrt{\frac{1}{n}\sum_{i=1}^{n}{∥x_{i}-{\hat{x}}_{\pi (i)}∥}_{2}^{2}}, \end{array}$$
  where $X=\{x_{1},x_{2},\cdots ,x_{m}\}$, $\tilde{X}=\left\{{\tilde{x}}_{1},{\tilde{x}}_{2},\cdots ,{\tilde{x}}_{n}\right\}$. $\Pi_{n}$ is the set of all permutations of {1,2,⋯,n}. $\pi \in \Pi_{n}$ can be written as π=(π(1),π(2),⋯,π(n)). ${∥x_{i}-{\hat{x}}_{\pi (i)}∥}_{2}^{2}={\left(x_{i}-{\hat{x}}_{\pi (i)}\right)}^{T}\left(x_{i}-{\hat{x}}_{\pi (i)}\right)$. The Wasserstein distance extends and provides a rigorous theoretical basis for a natural multitarget miss distance. However, it lacks a physically consistent interpretation when the sets have different cardinalities [52];
- OSPA distance:
  The OSPA was proposed to overcome the insensitive shortcoming, whose parameters can deal with the problem that the numbers of elements in the two sets do not match [53]. The OSPA metric between *X* and $\tilde{X}$ is:
  (7) $$d^{OSPA}\left(X,\tilde{X}\right)\overset{\Delta }{\;=}{\left(\frac{1}{n}\left(\min_{\pi \in \Pi_{n}}\sum_{i=1}^{m}d^{\left(c\right)}{\left(x_{i},{\tilde{x}}_{\pi (i)}\right)}^{p}+c^{p}\left(n-m\right)\right)\right)}^{\frac{1}{p}},$$
  where $d^{c}\left(x,\tilde{x}\right)$ is the cut-off distance between *x* and $\tilde{x}$ and $d^{c}\left(x,\tilde{x}\right)=\min\left\{d(x,\tilde{x}),c\right\}$, *c* denotes the truncation parameter, and *p* is the OSPA metric order parameter. The choice of parameters was given in [53]. The OSPA distance has been used widely in the literature [44,54,55,56] and has better properties for the multitarget error evaluation than the Hausdorff metric.
  In addition, there are various improved methods based on the OSPA [57], which are be enumerated as follows:
- Generalized OSPA (GOSPA):
  In the GOSPA metric, we look for an optimal assignment between the truth targets and the estimated tracks, leaving missed and false targets unsigned [58]. The GOSPA metric penalizes localization errors for properly detected targets, the NMT, and the NFT [59]. The GOSPA can be represented as an optimization over assignment sets:
  (8) $$d_{p}^{\left(c,2\right)}\left(X,\tilde{X}\right)={\left[\min_{\gamma \in \Gamma }\left(\sum_{\left(i,j\right)\in \gamma }d^{p}\left(x_{i},{\tilde{x}}_{j}\right)+\frac{c^{p}}{\alpha }\left(n+m-2\left|\gamma \right|\right)\right)\right]}^{\frac{1}{p}},$$
  where $\gamma \subseteq \left\{1,2,\cdots ,n\right\}\times \left\{1,2,\cdots ,m\right\}$, $\left(i,j\right),\left(i,j^{\prime }\right)\in \gamma \to j=j^{\prime }$, and $\left(i,j\right),\left(i^{\prime },j\right)\in \gamma \to i=i^{\prime }$. Γ denotes the set of all possible assignment sets γ. α is the additional parameter to control the cardinality mismatch penalty; in general, α =2. The terms $\frac{c^{p}}{\alpha }\left(m-\left|\gamma \right|\right)$ and $\frac{c^{p}}{\alpha }\left(n-\left|\gamma \right|\right)$ represent the costs (to the *p*th power) for the NMT and the NFT, respectively;
- OSPA-on-OSPA (OSPA)${}^{\left(2\right)}$metric:
  The OSPA${}^{\left(2\right)}$ metric [60,61] is the distance between two sets of tracks, which establishes an assignment between the real and the estimated trajectories that is not allowed to change with time and enables capturing the tracking errors of fragmentation and track switching. It is also simple to compute and flexible enough to capture many important aspects of the tracking performance. The OSPA${}^{\left(2\right)}$ distance is defined as follows:
  (9) $$d_{p,q}^{\left(c,2\right)}\left(X,\tilde{X};\omega \right)\overset{\Delta }{\;=}{\left(\frac{1}{n}\left(\min_{\pi \in \Pi_{n}}\sum_{i=1}^{m}d_{q}^{\left(c\right)}{\left(x_{i},{\tilde{x}}_{\pi (i)};\omega \right)}^{p}+c^{p}\left(n-m\right)\right)\right)}^{\frac{1}{p}},$$
  where *q* is the order of the base distance and *w* is a collection of convex weights.

Finally, we introduce the single integrated air picture (SIAP) metric. Despite the terminology, it is applicable to tracking in general and not just in relation to an air picture. It is made up of multiple individual metrics [62,63]. The SIAP metric requires an association between tracks and targets. We used a unique association in this paper. A description of the key metrics of the SIAP is given in Table 1.

## 3. CE Approaches

The above metrics provide the criteria for the PE of the tracking algorithm, which are combined to give the overall performance by the CE model. In this section, we review several CE approaches to analyze and judge the performance of target tracking.

### 3.1. The Weight of Each Evaluation Metric Set

The analytic hierarchy process (AHP) is given to ascertain the proportion of each metric, which was used to model and analyze the evaluation metric of the PE according to layers [64,65,66]. The specific steps are as follows.

**Step 1**  The assessment metric system:
  According to Section 2, all levels of evaluation metrics are established. $U_{i}\left(i\in \left[1,m\right]\right)$ in $U=\left\{U_{1},U_{2},\cdots ,U_{m}\right\}$ is the *i*th metric of the primary metric in the system; $U_{ij}\left(j\in \left[1,l\right]\right)$ in $U_{i}=\left\{U_{i1},U_{i2},\cdots ,U_{im}\right\}$ is the *j*th metric of $U_{i}$; $U_{ij}=\left\{U_{ij1},U_{ij2},\cdots ,U_{ijm}\right\}$, $U_{ijk}$ is the *k*th metric of $U_{ij}$. The rest can be established in the same manner;
**Step 2**  The comparison matrix $A_{ij}$:
  Citing the numbers 1-9 as a scale, each influencing metric $U_{i}$ in the above metric set *U* is determined according to the importance of the element to its corresponding quantized value $A_{ij}$. $A={\left(A_{ij}\right)}_{n\times n}$, $A_{ji}=\frac{1}{A_{ij}}$, $A_{ii}=1$;
**Step 3**  The maximum eigenvalue $\lambda_{max}$ of *A* and the corresponding normalized eigenvector *W*:
  *W* is denoted as $W=(W_{1},W_{2},\cdots W_{n})$, $\sum_{i=1}^{n}W_{i}=1$, where $W_{i}$ denotes the weight of the *i*th evaluation metric.

### 3.2. Cloud Barycenter Evaluation

Based on the traditional fuzzy set and probability theory, the cloud theory provides a powerful method by combining the qualitative information with the quantitative data [67]. As a kind of mathematical model, cloud theory describes the mapping relationship between quality and quantity through a fuzzy and stochastic relation completely [68]. The cloud barycenter evaluation developed from cloud theory is a CE method that has been extensively used in numerous complex systems, especially in the military field [67]. The cloud barycenter evaluation method is a qualitative and quantitative method to achieve the transformation between the conception and the data.

The cloud is represented by three digital characteristics, including the expected value $E_{x}$, the entropy $E_{n}$, and the hyper entropy $H_{e}$[67,69], where $E_{x}$ is the central value of the fuzzy concept in the defined domain, $E_{n}$ represents the degree of the fuzziness of the qualitative concept, and $H_{e}$ is the fuzzy measurement and the entropy of $E_{n}$, which is a mapping of the uncertainty of the qualitative concept.

**Figure 3:**
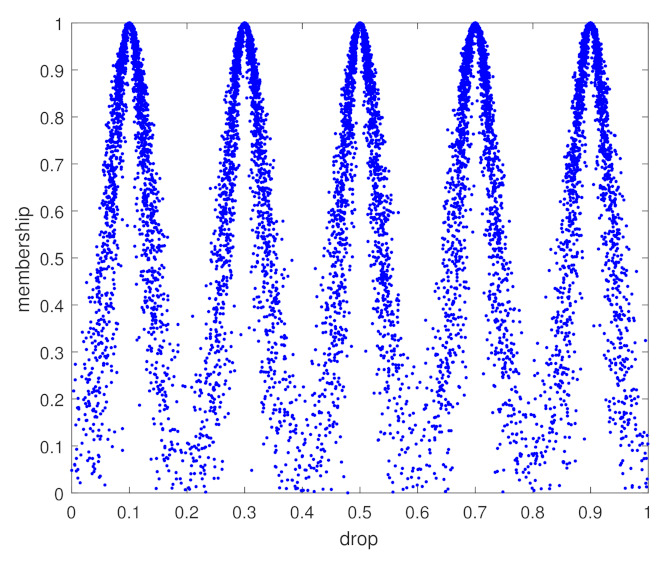
Qualitative evaluation of the cloud-generator model.

The cloud barycenter evaluation is realized by establishing the cloud model of each metric. The specific evaluation processes are as follows:

**Step 1**  The cloud model of the comment set:
  The comment set of metrics was ascertained by experts. For example, we set *S*= excellent, good, fair, worse, poor to denote the comment set of target tracking, which is shown in Table 2. We set the comment as the corresponding continuous number field interval [0,1]. The formula of the cloud model is represented as:
  (10) $$\left\{\begin{array}{c} E_{xi0}=\frac{c_{\min}+c_{\max}}{2},\\ E_{ni0}=\frac{c_{\max}-c_{\min}}{6}, \end{array}\right.$$
  where $E_{xi0}$, $E_{ni0}$ are the expected values and entropy of some qualitative comments, respectively.
**Step 2**  The quantitative and the qualitative variables for the given metric set;
  (a) The cloud model of quantitative metrics:
    The corresponding quantitative metrics values were established by *n* experts as $E_{x11},E_{x21},\cdots ,E_{xn1}$, which can be denoted by the cloud model:
    (11) $$E_{x1}=\frac{E_{x11}+E_{x21}+\cdots +E_{xn1}}{n},$$
    (12) $$E_{n1}=\frac{\max\left(E_{x11},E_{x21},\cdots ,E_{xn1}\right)-\min\left(E_{x11},E_{x21},\cdots ,E_{xn1}\right)}{6}.$$
  (b) The cloud model of qualitative metrics:
    In the same way, every qualitative metric, which are represented by the linguistic value, can also be described by the cloud model:
    (13) $$E_{x2}=\frac{E_{x12}E_{n12}+E_{x22}E_{n22}+\cdots +E_{xn2}E_{nn2}}{E_{n12}+E_{n22}+\cdots +E_{nn2}},$$
    (14) $$E_{n2}=E_{n12}+E_{n22}+\cdots +E_{nn2},$$
    where $E_{x12},E_{x22},\cdots ,E_{xn2}$ and $E_{n12},E_{n22},\cdots ,E_{nn2}$ denote the expected values and entropy of the cloud model, respectively;
**Step 3**  The weighted departure degree:
  $S=(S_{1},S_{2},\cdots ,S_{n})$ is the n-dimensional integrated barycenter vector, each dimension value of which is calculated by $S_{i}=g_{i}\times h_{i}\left(i=1,2,\cdots ,n\right)$, where $g_{i}=\left(E_{x1},E_{x2},\cdots ,E_{xn}\right)$ is the cloud barycenter position and $h_{i}=\left(W_{1},W_{2},\cdots ,W_{n}\right)$ is the cloud barycenter height calculated by the AHP. $S^{0}=\left({S_{1}}^{0},{S_{2}}^{0},\cdots ,{S_{n}}^{0}\right)$ denotes the ideal cloud vector. The synthesized vector is normalized as follows:
  (15) $$S_{i}^{T}=\left\{\begin{array}{c} \frac{S_{i}^{0}-S_{i}}{S_{i}^{0}},S_{i}<S_{i}^{0}.\\ \frac{S_{i}-S_{i}^{0}}{S_{i}^{0}},S_{i}\geq S_{i}^{0}. \end{array}\right.$$
  Finally, the weighted departure degree is given by:
  (16) $$\theta =\sum_{j=1}^{n}\left(S_{j}^{T}\times W_{j}\right)\left(0\leq \theta \leq 1\right);$$
**Step 4**  Result analysis:
  The comment set is put in a consecutive interval. Meanwhile, each comment value is realized by a cloud model. The cloud-generator model can be established as Figure 3 shows. The comment set can be divided into five categories: excellent, good, fair, worse, and poor. For a specific case, assessment results can be output by inputting 1+θ into the cloud-generator model.

### 3.3. Fuzzy CE Method

The fuzzy CE is based on fuzzy mathematics, which quantitatively expresses the objective attributes of some uncertain things [70,71,72]. The specific process is as follows:

**Step 1**  The metric set:
  We analyzed the result of target tracking and establish the evaluation metric set *U* as follows:
  (17) $$U=\left\{U_{1},U_{2},\cdots ,U_{n}\right\},$$
  where $U_{i}$ is the *i*th evaluation metric;
**Step 2**  The evaluation level set:
  The evaluation level set is given by $V=\{V_{1},V_{2},\cdots ,V_{m}\}$, where $V_{i}$ is the *i*th grey category. *V* is the remark collection, which is made up of remarks of the research object;
**Step 3**  The evaluation matrix:
  Starting from a single factor for the evaluation, we determine the degree of membership about evaluation objects to the evaluation level set and make the fuzzy evaluation. Then, combining the single-factor set, a multi-factor evaluation set is given by:
  (18) $$R=\left(\begin{array}{c} R_{1}\\ R_{2}\\ \cdots \\ R_{m} \end{array}\right)=\left(\begin{array}{cccc} r_{11} & r_{12} & \cdots & r_{1m}\\ r_{21} & r_{22} & \cdots & r_{2m}\\ \cdots & \cdots & \cdots & \cdots \\ r_{n1} & r_{n2} & \cdots & r_{nm} \end{array}\right),$$
  where $r_{ij}$ denotes the membership degree of $U_{i}$ corresponding to $V_{j}$;
**Step 4**  The fuzzy CE value:
  (19) $$\begin{array}{cc} C=BR & =\left(\begin{array}{cccc} b_{1} & b_{2} & \cdots & b_{n} \end{array}\right)\left(\begin{array}{cccc} r_{11} & r_{12} & \cdots & r_{1m}\\ r_{21} & r_{22} & \cdots & r_{2m}\\ \cdots & \cdots & \cdots & \cdots \\ r_{n1} & r_{n2} & \cdots & r_{nm} \end{array}\right)=\left(\begin{array}{cccc} c_{1} & c_{2} & \cdots & c_{j} \end{array}\right), \end{array}$$
  where *C* is the fuzzy CE set and *B* is the weight of the metric.
  According to the principle of the maximum membership degree, the comprehensive value of the PE is obtained; thereby, the corresponding performance levels [70] are calculated.

### 3.4. Grey Clustering

Grey theory is a useful methodology for incomplete information systems. Grey relational analysis can be used to analyze relationships between the uncertainty and the gray category [73,74]. The main steps of the method are as follows:

**Step 1**  Triangular whitenization weight functions are established and obtained as follows:
  (20) $$f_{1j}^{k}\left(d_{ij}\right)=f_{1j}^{k}\left(c_{1j}^{k},\infty \right)=\left\{\begin{array}{c} \frac{d_{ij}}{c_{1j}^{k}},d_{ij}\in \left[0,c_{1j}^{k}\right]\\ 1,d_{ij}\in \left(c_{1j}^{k},\infty \right)\\ 0,d_{ij}\notin \left[0,\infty \right) \end{array}\right.,$$
  (21) $$f_{2j}^{k}\left(d_{ij}\right)=f_{2j}^{k}\left(-,c_{2j}^{k},+\right)=\left\{\begin{array}{c} \frac{d_{ij}}{c_{2j}^{k}},d_{ij}\in \left[0,c_{2j}^{k}\right]\\ \frac{d_{ij}-2c_{2j}^{k}}{-c_{2j}^{k}},d_{ij}\in \left(c_{2j}^{k},2c_{2j}^{k}\right]\\ 0,d_{ij}\notin \left[0,2c_{2j}^{k}\right] \end{array}\right.,$$
  (22) $$f_{3j}^{k}\left(d_{ij}\right)=f_{3j}^{k}\left(0,c_{3j}^{k}\right)=\left\{\begin{array}{c} 1,d_{ij}\in \left[0,c_{3j}^{k}\right]\\ \frac{d_{ij}-2c_{3j}^{k}}{-c_{3j}^{k}},d_{ij}\in \left(c_{3j}^{k},2c_{3j}^{k}\right]\\ 0,d_{ij}\notin \left[0,2c_{3j}^{k}\right] \end{array}\right.,$$
  where $d_{ij}\left(i=1,2,\cdots ,n;j=1,2,\cdots ,m\right)$ is the sample of the *i*th algorithm about *j*th evaluation metric and $c_{j}^{k}$ is the midpoints of the *j*th clustering metric belonging to the *k*th grey category. The type of measure determines the choice of three functions: If the measure is extremely large data (preferably larger), we select $f_{1j}^{k}\left(d_{ij}\right)$. If it is the moderate measure, $f_{2j}^{k}\left(d_{ij}\right)$ is selected. If it is extremely small, then $f_{3j}^{k}\left(d_{ij}\right)$ is the first choice;
**Step 2**  The clustering coefficient:
  (23) $$\sigma_{i}^{k}=\sum_{j=1}^{m}f_{j}^{k}\left(d_{ij}\right)w_{j},$$
  (24) $$\delta_{i}^{k}=\frac{\sigma_{i}^{k}}{\sum_{k=1}^{s}\sigma_{i}^{k}},$$
  where $w_{j}$ is the weight of the *j*th clustering and is determined by the AHP. $\sigma_{i}^{k}$ is the weight cluster coefficient of the *i*th algorithm about the *k*th grey category, and $\delta_{i}^{k}$ is the normalized weight cluster coefficient, respectively [75];
**Step 3**  The integrated clustering coefficient $\eta_{i}$ for each monitoring point with respect to the grey classes *k* can be calculated with the following equation [76]:
  (25) $$\eta_{i}=\sum_{k=1}^{s}k\cdot \delta_{i}^{k},\left(i=1,2,\cdots ,n\right);$$
**Step 4**  According to the integrated clustering coefficient, the evaluation result is determined. The value range of the integrated clustering coefficient is divided into *s* intervals of the same length, which are: [1,1+s−1/s], [1+s−1/s,1+2(s−1)/s], ⋯, [s−s−1/s,s]. The track algorithm is judged as the *k*th grey category, when $\eta_{i}$ belongs to $\left[1+(k-1)(s-1)/s,1+k(s-1)/s\right]$.

## 4. Rating and Overall Performance

Most simulations are run in the Monte Carlo scenario to describe the characteristics of the performance metrics. In [21], the analysis and assessment of the tracking algorithm were performed both with simulated and real data, where the real data were measured with the Multi-Static Primary Surveillance Radar (MSPSR) L-Band demonstrator, and the metrics were calculated for the performance evaluation such as the mean variance statistic of the NMT, RTF, RMSE, etc. Refs. [25,77] calculated the GOSPA varying values of *c* and γ using a multiple target tracking example in the MATLAB code. The COCO 2017 validation set and the MOTChallenge (MOT17) dataset were used in terms of the Hausdorff, Wasserstein, and OSPA metrics [78]. In the paper, the metrics mentioned in Section 2 were combined to give a score or a membership value by the aforementioned CE approaches. Three measures were taken together to judge the efficiency of target tracking in the cloud barycenter evaluation (the synthetic measures do not involve simulations). At the same time, the realization of the fuzzy theory and the grey clustering is in the absence of the correctness and the timeliness scenario. In order to demonstrate it more vividly, our simulation was performed by using a graphical user interface (GUI).

### 4.1. Application of Cloud Theory for Target Tracking

**Figure 4:**
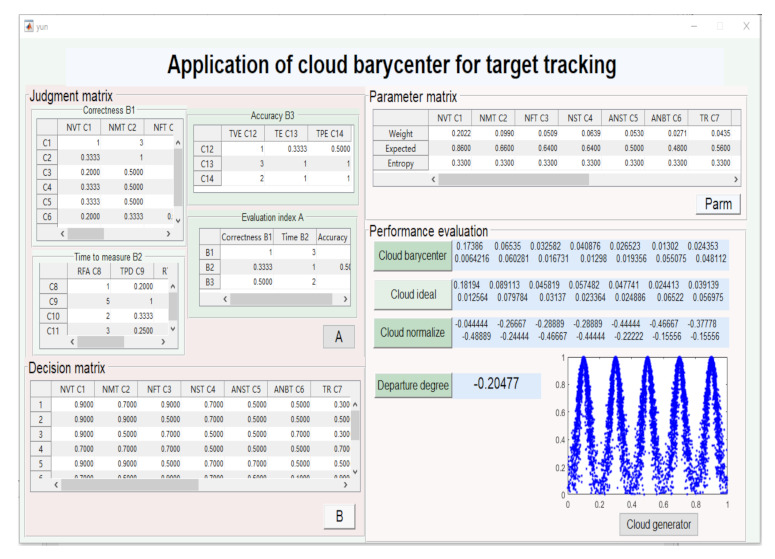
PE of cloud gravity for target tracking.

In this section, we discuss the application of cloud theory for target tracking. In this scenario, three categories of the performance metrics are involved, and the last is the accuracy, which is divided into the TE, TPE, and TVE. The judgment matrix was given by experts and displayed in Figure 4, and then, the metric weights were ascertained. $W_{1}$ = (0.5436, 0.1634, 0.2970) is the weight of three primary metrics. Then, $W_{2}$ = (0.3746, 0.1835, 0.0943, 0.1184, 0.0983, 0.0503, 0.0806) is the weight of the correctness measure; $W_{3}$ = (0.0854, 0.5424, 0.2133, 0.1588) is the weight of the timeliness measure; $W_{4}$ = (0.1692, 0.4434, 0.3874) is the weight of the accuracy measure. We can draw some conclusions in which the tracking accuracy and correctness are more important than the timeliness in judging the efficiency of target tracking, where the sequential ratio of those is 0.2970, 0.5346, and 0.1634.

According to the evaluation of experts, *S* = $\left\{\mathrm{excellent},\mathrm{good},\mathrm{fair},\mathrm{worse},\mathrm{poor}\right\}$ was placed in [0,1]. Therefore, Table 3 represents the results of the cloud model of comments.

$$S=\left[\begin{array}{cccccccccccccc} 0.9 & 0.7 & 0.9 & 0.7 & 0.5 & 0.5 & 0.3 & 0.3 & 0.5 & 0.3 & 0.1 & 0.7 & 0.7 & 0.7\\ 0.9 & 0.9 & 0.5 & 0.7 & 0.5 & 0.5 & 0.5 & 0.3 & 0.5 & 0.1 & 0.3 & 0.5 & 0.5 & 0.5\\ 0.9 & 0.5 & 0.7 & 0.5 & 0.5 & 0.7 & 0.3 & 0.5 & 0.9 & 0.5 & 0.5 & 0.7 & 0.7 & 0.7\\ 0.7 & 0.7 & 0.7 & 0.5 & 0.5 & 0.5 & 0.7 & 0.7 & 0.7 & 0.7 & 0.5 & 0.5 & 0.9 & 0.7\\ 0.9 & 0.9 & 0.5 & 0.7 & 0.7 & 0.5 & 0.5 & 0.3 & 0.5 & 0.5 & 0.7 & 0.9 & 0.9 & 0.9\\ 0.7 & 0.5 & 0.9 & 0.7 & 0.5 & 0.1 & 0.9 & 0.1 & 0.9 & 0.9 & 0.3 & 0.5 & 0.9 & 0.7\\ 0.9 & 0.7 & 0.5 & 0.7 & 0.3 & 0.7 & 0.5 & 0.7 & 0.7 & 0.7 & 0.5 & 0.9 & 0.7 & 0.9\\ 0.9 & 0.7 & 0.5 & 0.5 & 0.3 & 0.5 & 0.7 & 0.7 & 0.3 & 0.3 & 0.5 & 0.7 & 0.7 & 0.7\\ 0.9 & 0.5 & 0.7 & 0.7 & 0.3 & 0.3 & 0.5 & 0.5 & 0.3 & 0.3 & 0.9 & 0.9 & 0.9 & 0.9\\ 0.9 & 0.5 & 0.5 & 0.7 & 0.9 & 0.5 & 0.7 & 0.5 & 0.5 & 0.5 & 0.7 & 0.7 & 0.7 & 0.9 \end{array}\right]$$

*S* is the decision matrix, which denotes the integrated cloud gravity center. Combining Equations (13) and (14) and Table 3, the cloud model of the parameter status is calculated and given by Table 4.

Then, the conclusion can be obtained in the part of “performance evaluation”. *W* = [0.2022, 0.0990, 0.0509, 0.0639, 0.0530, 0.0271, 0.0435, 0.0140, 0.0886, 0.0349, 0.0260, 0.0277, 0.0725, 0.0633]. In the ideal state, *E* = [0.9, 0.9, 0.9, 0.9, 0.9, 0.9, 0.9, 0.9, 0.9, 0.9, 0.9, 0.9, 0.9, 0.9]; the integrated evaluation vector of the cloud barycenter is $S^{0}=W\times E=$ (0.1819, 0.0891, 0.0458, 0.0575, 0.0477, 0.0244, 0.0391, 0.0126, 0.0798, 0.0314, 0.0234, 0.0249, 0.0652, 0.0570). The actual integrated vector is *S* = $\left(W_{1},W_{2},\cdots ,W_{14}\right)\times \left(E_{x1},E_{x2},\cdots ,E_{x14}\right)$ = (0.1739, 0.0653, 0.0326, 0.0409, 0.0265, 0.0130, 0.0244, 0.0064, 0.0603, 0.0167, 0.0130, 0.0194, 0.0551, 0.0481). *S* is normalized as $S^{T}=\left(S_{1}^{T},S_{2}^{T},\cdots ,S_{14}^{T}\right)=$ (−0.0444, −0.2667, −0.2889, −0.2889, −0.4444, −0.4667, −0.3778, −0.4889, −0.2444, −0.4667, −0.4444, −0.2222, −0.1556, −0.1556). Finally, the weighted departure degree can be acquired as θ = −0.20477.

In the ideal state, θ for PE is zero; however, the actual θ is −0.20477. When 1+θ=0.79523 is input into the cloud generator model, the cloud drops close to the “good” cloud object. Then, the PE result of target tracking is deemed to be good. We came up with a conjecture that the measure of the accuracy and the correctness are more effective than the timeliness in target tracking. Then, we verified it in the following simulation.

### 4.2. Application of Fuzzy CE for Target Tracking

Fuzzy CE uses the fuzzy mathematics tool to depict the influence of various metrics, which was applied in the situation without the timeliness measure. Here, *U*= NMT $U_{1}$, NVT $U_{2}$, NST $U_{3}$, NFT $U_{4}$, TSE $U_{5}$, TBE $U_{6}$, TE $U_{7}$. *B* = (0.1621, 0.1284, 0.0773, 0.1120, 0.0836, 0.2000, 0.2365) is the weight of the seven metrics. $V=\left\{V_{1},V_{2},V_{3},V_{4}\right\}$ denotes the comment set, corresponding four levels: “excellent”, “good”, “medium”, and “poor”. Here, the subordinate degree was determined by the expert assessment. We obtain R,C as follows.

$$R=\left[\begin{array}{c} R1\\ R2\\ R3\\ R4\\ R5\\ R6\\ R7 \end{array}\right]=\left[\begin{array}{cccc} 0.2 & 0.4 & 0.4 & 0\\ 0.3 & 0.4 & 0.3 & 0\\ 0.1 & 0.4 & 0.5 & 0\\ 0.2 & 0.4 & 0.4 & 0\\ 0.1 & 0.5 & 0.4 & 0\\ 0.2 & 0.3 & 0.5 & 0\\ 0 & 0.5 & 0.5 & 0 \end{array}\right]$$

$$\begin{array}{c} \begin{array}{c} C=BR={\left[\begin{array}{c} 0.1621\\ 0.1284\\ 0.0773\\ 0.1120\\ 0.2000\\ 0.1922\\ 0.2365 \end{array}\right]}^{T}\left[\begin{array}{cccc} 0.2 & 0.4 & 0.4 & 0\\ 0.3 & 0.4 & 0.3 & 0\\ 0.1 & 0.4 & 0.5 & 0\\ 0.2 & 0.4 & 0.4 & 0\\ 0.1 & 0.5 & 0.4 & 0\\ 0.2 & 0.3 & 0.5 & 0\\ 0 & 0.5 & 0.5 & 0 \end{array}\right]=\left[\begin{array}{cccc} 0.1494 & 0.4120 & 0.4386 & 0 \end{array}\right] \end{array} \end{array}$$

**Figure 5:**
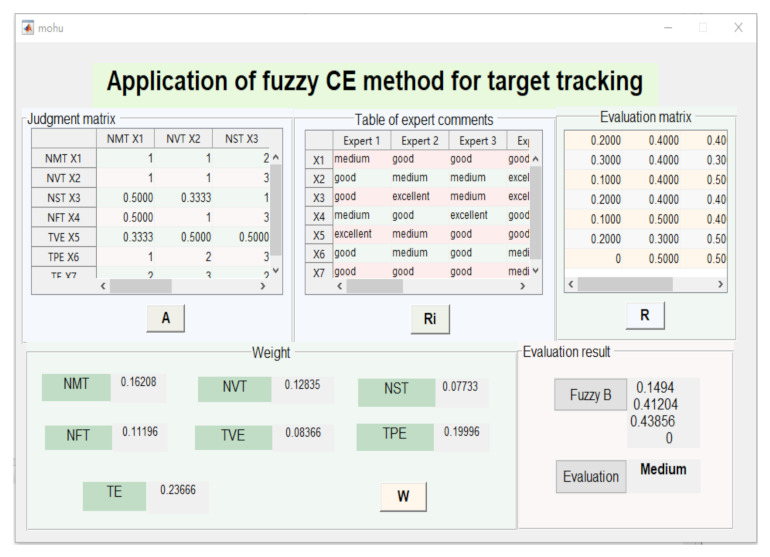
PE of target tracking based on fuzzy CE.

The simulation result is given in Figure 5. According to the metric information, we could obtain the fuzzy CE of all the evaluation information. The fuzzy CE set was calculated and performed the “medium” in terms of the maximum membership degree on the basis of the principle. To combine the analysis above, the total score of the PE for target tracking can be given by: D = 0.1494 × 90 + 0.4120 × 80 + 0.4386 × 70 = 77.108, and the result showed that the performance for target tracking performed medium.

### 4.3. PE in Target Tracking Using Grey Clustering

In this section, representative metrics of the timeliness and the accuracy measures were chosen, which included the TPE, TL, TVE, TPD, and RFA. *w* = 0.29783, 0.088788, 0.15777, 0.29783, 0.15777 is the weight of these. Four grey categories were established, where *K* = $\left\{1,2,3,4\right\}$ denotes “excellent”, “good”, “medium”, and “poor”. Based on the scenario [79], the following tracking methods were used for the performance evaluation where results were available for 200 Monte Carlo runs:

(1) Pseudo-observation (PS) [80];
(2) Projection (PRO) [81,82];
(3) Karush–Kuhn–Tucker (KKT) [83];
(4) Karush–Kuhn–Tucker–Kalman filter (KKT_KF) [79];
(5) Unconstrained Kalman filter (UKF) [84];
(6) Trajectory function of time (T-FoT) [85,86].

Performance metrics were now found based on the given tracking results, which are given in Table 5. According to Table 5, we can determine the whitenization weight functions in Table 6. Taking the first metric (TPE) as an example, the four whitenization weight functions are:

$$f_{1}^{1}\left(6,\infty \right)=\left\{\begin{array}{c} \frac{d_{ij}}{6},d_{ij}\in \left[0,6\right]\\ 1,d_{ij}\in \left(6,\infty \right)\\ 0,d_{ij}\notin [0,\infty ) \end{array}\right.$$

$$f_{1}^{2}\left(-,4.5,+\right)=\left\{\begin{array}{c} \frac{d_{ij}}{c_{2j}^{k}},d_{ij}\in \left[0,4.5\right]\\ \frac{d_{ij}-9}{-4.5},d_{ij}\in \left(4.5,9\right]\\ 0,d_{ij}\notin \left[0,9\right] \end{array}\right.$$

$$f_{1}^{3}\left(-,3,+\right)=\left\{\begin{array}{c} \frac{d_{ij}}{3},d_{ij}\in \left[0,3\right]\\ \frac{d_{ij}-6}{-3},d_{ij}\in \left(3,6\right]\\ 0,d_{ij}\notin \left[0,6\right] \end{array}\right.$$

$$f_{1}^{4}\left(0,1\right)=\left\{\begin{array}{c} 1,d_{ij}\in \left[0,1\right]\\ \frac{d_{ij}-2}{-1},d_{ij}\in \left(1,2\right]\\ 0,d_{ij}\notin \left[0,2\right] \end{array}\right.$$

$f_{1j}^{k}\left(c_{1j}^{k},\infty \right)$ was applied in the excellent grey category, which is an upper measure. The good grey category and medium grey category were selected by $f_{2j}^{k}\left(-,c_{2j}^{k},+\right)$. $f_{3j}^{k}\left(0,c_{3j}^{k}\right)$ is a whitenization weight function of lower measure, which was used in the poor grey category next. From the weight of the evaluation metric and the grey clustering, the weighted clustering coefficient matrix σ can be determined.

$$\sigma =\left(\sigma_{i}^{k}\right)=\left|\begin{array}{cccc} 0.7331 & 0.8083 & 0.4698 & 0\\ 0.8656 & 0.8006 & 0.3427 & 0\\ 0.9255 & 0.7552 & 0.2425 & 0\\ 0.6936 & 0.8847 & 0.5296 & 0\\ 0.8024 & 0.8481 & 0.2258 & 0\\ 0.8966 & 0.6882 & 0.1425 & 0 \end{array}\right|$$

Furthermore, the normalized clustering coefficient matrix can be obtained:

$$\delta_{i}^{k}=\left|\begin{array}{cccc} 0.3645 & 0.4019 & 0.2336 & 0\\ 0.4309 & 0.3985 & 0.1706 & 0\\ 0.4813 & 0.3927 & 0.1261 & 0\\ 0.3290 & 0.4197 & 0.2513 & 0\\ 0.4277 & 0.4520 & 0.1203 & 0\\ 0.5191 & 0.3984 & 0.0825 & 0 \end{array}\right|$$

**Figure 6:**
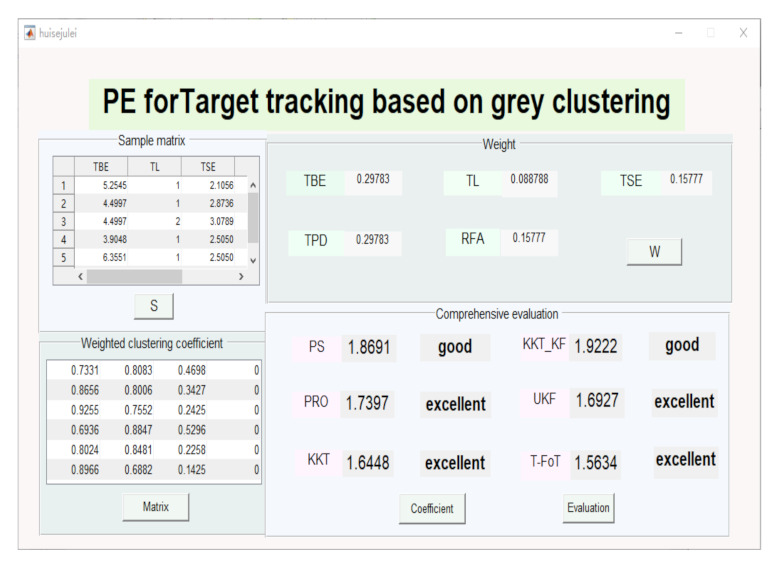
PE for target tracking based on grey clustering.

The simulation result is shown in Figure 6. The integrated clustering coefficients of the six algorithms were calculated. For example, the integrated clustering coefficient of the PS in the first algorithm was $\eta_{1}$ = $\sum_{k=1}^{4}k\cdot \delta_{i}^{k}$ = 1×0.3645 + 2×0.4019 + 3×0.2336 + 4×0 = 1.8691, and the other coefficients were $\eta_{2}$ = 1.7397 (PRO), $\eta_{3}$ = 1.6448 (KKT), $\eta_{4}$ = 1.9222 (KKT_KF), $\eta_{5}$ = 1.6927 (UKF), $\eta_{6}$ = 1.5634 (T-FoT), respectively. The value range of the integrated clustering coefficient was divided into four intervals of the same length. According to **Step 4** in Section 3.4, $\eta_{2},\eta_{3},\eta_{5},\eta_{6}\in \left[1,1+3/4\right]$, $\eta_{1},\eta_{4}\in \left[1+3/4,1+6/4\right]$, and the evaluated result was that the methods of the PRO, KKT, UKF, and T-FoT had “excellent” performance and the methods of the PS and KKT_KF “good” performance. It is not difficult to find that the CE method for target tracking achieved a satisfactory result by comparing the above algorithms.

The simulation showed that the three measures contained sufficient information for evaluation by the comparative analysis. However, there were slight differences in the scenarios in which the one lacks correctness and the other one lacks timeliness. The results of grey clustering were consistent with cloud theory, and the fuzzy CE showed poor results. In other words, for this case, it can be concluded that the timeliness measure was less informative than the correctness and accuracy measures.

So far, on the one hand, PE has a tendency to focus on improving OSPA. On the other hand, PE metrics have been redefined using different association algorithms. A more effective PE method needs to be explored to enhance the algorithm efficiency.

## 5. Conclusions and Remaining Challenges

This paper reviewed PE metrics for target tracking and some CE approaches. The measures were divided into three categories and described for each category. Finally, the simulation result showed that a combination of metrics from different classes can provide a criterion for PE in target tracking.

Instead of estimating the discrete-time state of the target, it is actually of greater interest to estimate the continuous-time trajectory of the target, which contains more information than the discrete time series of point estimates. The T-FoT framework [85,87,88] is promising and powerful as it completely describes the movement pattern/dynamic behavior of the targets over time and enables the use of many curve-fitting tools such as the Gauss process and neural networks, in addition to various flexible parametric regression analysis methods. However, based on this framework, the output of the estimator or tracker is a spatio-temporal trajectory function, or functions in the case of multiple targets. How to evaluate the quality of the trajectory functions/curves in the presence of missing detection and false alarms remains an open problem.

## Figures and Tables

**Table 1:** The key metrics of the SIAP.

Metric	Description
Ambiguity	A measure of the number of tracks assigned to each true object
Completeness	The percentage of live objects with tracks on them
LS	The percentage of time spent tracking true objects across the dataset
LT	1/R, where R is the average number of excess tracks assigned;
	the higher this value, the better
Positional Accuracy	Given by the average positional error of the track to the truth
Spuriousness	The percentage of tracks unsigned to any object
Velocity Accuracy	The average error in the velocity of the track to the truth
Number of Targets	The total number of targets
Number of Tracks	The total number of tracks

**Table 2:** Number field variation interval of the comments set.

Comments	Excellent	Good	Fair	Worse	Poor
Number field interval	[1,c1]	[c1,c2]	[c2,c3]	[c3,c4]	[c4,0]

**Table 3:** Cloud model of the comments.

Comments	Number Field Interval	Numeral Characteristics
excellent	[1,0.8]	(0.9,0.033)
good	[0.8,0.6]	(0.7,0.033)
fair	[0.6,0.4]	(0.5,0.033)
worse	[0.4,0.2]	(0.3,0.033)
poor	[0.2,0]	(0.1,0.033)

**Table 4:** The cloud model of the parameter status.

Parameter	Expectations	Entropy
C1	0.86	0.33
C2	0.66	0.33
C3	0.64	0.33
C4	0.64	0.33
C5	0.5	0.33
C6	0.48	0.33
C7	0.56	0.33
C8	0.46	0.33
C9	0.68	0.33
C10	0.48	0.33
C11	0.5	0.33
C12	0.7	0.33
C13	0.76	0.33
C14	0.76	0.33

**Table 5:** Metric statistics of each tracking algorithm.

Arithmetic	TPE	TL	TVE	TPD	RFA
PS	5.2545	1	2.1056	0.958	0.00042
PRO	4.4997	1	2.8736	0.973	0.0085
KKT	4.4997	2	3.0789	0.965	0.014
KKT_KF	3.9048	1	2.505	0.966	0.0007
UKF	6.3551	1	2.505	0.968	0.001
T-FoT	5.5309	2	3.0789	0.961	0.005

**Table 6:** Whitenization weight functions of four grey categories.

Excellent Grey Category	Good Grey Category
$f_{1}^{1}\left(c_{1}^{1},\infty \right)=f_{1}^{1}\left(6,\infty \right)$	$f_{1}^{2}\left(-,c_{1}^{2},+\right)=f_{1}^{2}\left(-,4.5,+\right)$
$f_{2}^{1}\left(c_{2}^{1},\infty \right)=f_{2}^{1}\left(1.5,\infty \right)$	$f_{2}^{2}\left(-,c_{2}^{2},+\right)=f_{2}^{2}\left(-,1.25,+\right)$
$f_{3}^{1}\left(c_{3}^{1},\infty \right)=f_{3}^{1}\left(3,\infty \right)$	$f_{3}^{2}\left(-,c_{3}^{2},+\right)=f_{3}^{2}\left(-,2.6,+\right)$
$f_{4}^{1}\left(c_{4}^{1},\infty \right)=f_{4}^{1}\left(0.965,\infty \right)$	$f_{4}^{2}\left(-,c_{4}^{2},+\right)=f_{4}^{2}\left(-,0.95,+\right)$
$f_{5}^{1}\left(c_{5}^{1},\infty \right)=f_{5}^{1}\left(0.01,\infty \right)$	$f_{5}^{2}\left(-,c_{5}^{2},+\right)=f_{5}^{2}\left(-,0.001,+\right)$
**Medium Grey Category**	**Poor Grey Category**
$f_{1}^{3}\left(-,c_{1}^{3},+\right)=f_{1}^{3}\left(-,3,+\right)$	$f_{1}^{4}\left(0,c_{1}^{4}\right)=f_{1}^{4}\left(0,1\right)$
$f_{2}^{3}\left(-,c_{2}^{3},+\right)=f_{2}^{3}\left(-,1,+\right)$	$f_{2}^{4}\left(0,c_{2}^{4}\right)=f_{2}^{4}\left(0,0.5\right)$
$f_{3}^{3}\left(-,c_{3}^{3},+\right)=f_{3}^{3}\left(-,2,+\right)$	$f_{3}^{4}\left(0,c_{3}^{4}\right)=f_{3}^{4}\left(0,1\right)$
$f_{4}^{3}\left(-,c_{4}^{3},+\right)=f_{4}^{3}\left(-,0.5,+\right)$	$f_{4}^{4}\left(0,c_{4}^{4}\right)=f_{4}^{4}\left(0,0.5\right)$
$f_{5}^{3}\left(-,c_{5}^{3},+\right)=f_{5}^{3}\left(-,0.0005,+\right)$	$f_{5}^{4}\left(0,c_{5}^{4}\right)=f_{5}^{4}\left(0,0.0001\right)$

## Data Availability

Data will be made available upon request.

## Abbreviations

The following abbreviations are used in this manuscript:

CE	Comprehensive evaluation
PE	Performance evaluation
TPE	Track position error
TE	Trajectory error
TVE	Track velocity error
NVT	Number of valid tracks
NMT	Number of missed targets
NFT	Number of false tracks
NST	Number of spurious tracks
ANST	Average number of swaps in tracks
ANBT	Average number of broken tracks
TR	Tracks redundancy
RFA	Rate of false alarms
TPD	Track probability of detection
RTF	Rate of track fragmentation
TL	Track latency
TET	Total execution time
OSPA	Optimal subpattern assignment
AHP	Analytic hierarchy process
SIAP	Single integrated air picture
MOPT	Multiple-object-tracking precision
MOTA	Multiple-object-tracking accuracy
GUI	Graphical user interface
PS	Pseudo-observation
PRO	Projection
KKT	Karush–Kuhn–Tucker
KKT_KF	Karush–Kuhn–Tucker–Kalman filter
UKF	Unconstrained Kalman filter
T-FoT	Trajectory function of time
